# Fine‐mapping of existing and novel functional quantitative trait locireveals local genetic influences of epigenetic and transcriptomic regulation in human brains

**DOI:** 10.1002/alz.092049

**Published:** 2025-01-03

**Authors:** Edoardo Marcora, Hao Sun

**Affiliations:** ^1^ Ronald M. Loeb Center for Alzheimer’s disease, New York, NY USA; ^2^ Icahn School of Medicine at Mount Sinai, New York, NY USA

## Abstract

**Background:**

The FunGen‐xQTL project has significantly advanced genetics by developing and exploring novel quantitative trait loci (QTL) types in human brains, enriching our understanding of complex neurological disease etiology. We broadened the scope of epigenomic QTL analysis, integrating histone acetylation QTLs (haQTLs) and methylation QTLs (mQTLs) that affect multiple histone acetylation peaks or methylation CpG sites spatially. Additionally, we investigated a new category of splicing QTLs (sQTLs) implicated in nonsense‐mediated decay (NMD).

**Method:**

Utilizing unique computational methods developed by FunGen‐xQTL for genome‐wide statistical fine‐mapping, we dissected intricate gene regulation networks at single‐base resolution, effectively accounting for linkage disequilibrium confounding.

**Result:**

Our research has unveiled complex multi‐omic regulations via fine‐mapped epigenomic QTLs using our new method called fSuSiE. We identified specific genetic variants performing dual roles as both epigenomic and eQTLs across different genes, indicative of 3D multi‐gene interactions. For instance, cis‐variant rs62324750 for *TMEM131L* uniquely acts as a negative eQTL, negative haQTL, and positive mQTL. This complex interaction extends to nearby genes *TRIM2* and *MND1*, where rs11728062 impacts gene expression as a negative haQTL and positive mQTL through epigenomic markers spanning gene bodies. Oligodendrocyte‐specific Hi‐C data further support these findings, suggesting novel regulatory mechanisms involving disruption of transcriptional repressor bindings. Moreover, neuronal Hi‐C and ChiP‐Seq data uncover a unique xQTL interaction pattern in *APOL2*, indicative of 3D protein‐chromatin structures in transcription regulation.

Employing LeafCutter2 methods from FunGen‐xQTL, we investigated the genetic basis of NMD by identifying premature termination codons from RNA‐seq data and fine‐map the corresponding sQTL for such unproductive splicing events. In the ROSMAP DLPFC dataset, we distinguished 45,999 unproductive from 130,664 productive splicing events for NMD. We observed a higher colocalization ratio of unproductive sQTLs with eQTLs, illuminating new insights into AD etiology. Notably, an unproductive sQTL in *TSPAN14*, colocalized with an AD GWAS signal, revealed significant gene expression and intron usage differences across genotypes, suggesting RNA expression of *TSPAN14* may be mediated by genetically regulated NMD events.

**Conclusion:**

Our work demonstrates the complex interplay of novel QTL types in gene regulation and offers significant insights for future research into the genetic mechanisms of complex diseases.

